# Supplementary material for: Peripheral and Central Iron Measures in Alcohol Use Disorder and Aging: A Quantitative Susceptibility Mapping Pilot Study
Source: Int J Mol Sci. 2023 Feb 24;24(5):4461. doi: 10.3390/ijms24054461 (PMC10002495; doi:10.3390/ijms24054461)
Supplement: Supplementary file 1 [file ijms-24-04461-s001.zip › ijms-2232898-supplementary.pdf]

## Peripheral and Central Iron Measures in Alcohol Use Disorder and Aging: A Quantitative Susceptibility Mapping Pilot Study

Aiden R. Adams [1], Xinyi Li [1], Juliana I. Byanyima [1], Sianneh A. Vesslee [1], Thanh D. Nguyen [2], Yi Wang [2], Brianna Moon [3], Timothy Pond [1], Henry R. Kranzler [1], Walter R. Witschey [3], Zhenhao Shi [1,†] and Corinde E. Wiers [1,3,†,*]

[1] Center for Studies of Addiction, Department of Psychiatry, Perelman School of Medicine, University of Pennsylvania, 3535 Market St Ste 500, Philadelphia, PA 19104, USA;
[2] Department of Radiology, Weill Cornell Medicine, 525 E 68th St, New York, NY 10065, USA;
[3] Department of Radiology, Perelman School of Medicine, University of Pennsylvania, 3400 Civic Center Blvd, South Pavilion, Room 11-155, Philadelphia, PA 19104, USA;
[*] Correspondence: corinde.wiers@pennmedicine.upenn.edu; Tel.: +1-215-746-1898
[†] These authors contributed equally to this work.

## Supplementary Material

**Table S1.** Summary of regression analysis for serum ferritin from the National Institutes of Health (NIH) sample in n=583 inpatients with AUD and n=470 healthy controls

| | Serum Ferritin (NIH) | | |
|---|---|---|---|
| *Variable* | *B* | *SE B* | *β* |
| AUD | 123.7 | 15.05 | **0.256*** ** |
| Age | 1.476 | 0.566 | **0.080** ** |
| BMI | -3.250 | 1.406 | **-0.068*** |
| Sex | -108.4 | 14.48 | **-0.221*** ** |
| CRP | 2.166 | 0.092 | **0.092** ** |
| $R^2$ | 0.173 | | |
| *F* | **42.70*** ** | | |

Abbreviations: AUD alcohol use disorder, BMI body mass index, SE standard error. CRP C-reactive protein. Dummy coding: AUD (1=AUD, 0=healthy control); sex (1=female, 0=male).
*p<0.05, **p<0.01, ***p<0.001

**Table S2.** Summary of regression analysis for serum ferritin from the University of Pennsylvania (Penn) sample in n=10 participants with AUD and n=8 healthy controls

| | Serum Ferritin (Penn) | | |
|---|---|---|---|
| *Variable* | *B* | *SE B* | *β* |
| AUD | 67.28 | 31.12 | **0.523*** |
| Age | -1.661 | 1.063 | -0.325 |
| BMI | 0.562 | 3.493 | 0.040 |
| Sex | -31.91 | 28.67 | -0.243 |
| $R^2$ | 0.474 | | |
| *F* | **2.934^** | | |

Abbreviations: AUD alcohol use disorder, BMI body mass index, SE standard error. Dummy coding: AUD (1=AUD, 0=healthy control); sex (1=female, 0=male).
^p<0.06, *p<0.05

**Table S3**. Iron panel results (mean and standard deviation) from the University of Pennsylvania (Penn) sample

| Characteristics | AUD (n=10) | HC (n=8) | *p* value |
|---|---|---|---|
| Ferritin | 119.4 (67.4) | 51.0 (41.0) | 0.023 |
| TIBC | 316.0 (23.0) | 324.3 (69.8) | 0.37 |
| UIBC | 193.6 (54.9) | 227.1 (90.4) | 0.35 |
| Iron | 122.4 (39.6) | 97.1 (56.3) | 0.28 |
| Iron Saturation | 38.4 (15.5) | 31.4 (18.4) | 0.39 |
| Transferrin | 266.5 (24.5) | 267.7 (55.5) | 0.95 |

Abbreviations: AUD alcohol use disorder, TBIC Total iron-binding capacity, UIBC unsaturated iron-binding capacity

While our QSM study was underpowered to perform a genetic analysis of *HFE*, we analyzed the effects of *HFE* rs1799945 genotype on serum ferritin and its interaction with AUD status in the NIH sample (n= 484 AUD and n=431 controls for whom genetic data were available), as per Kroll et al, 2022 [7]. *HFE* SNP rs1799945 was determined using the Illumina human OmniExpressExome array (Illumina, San Diego, CA, USA). To correct for genetic ancestry, a principal component analysis (PCA) was performed based on 133,486 SNPs using the -CPA option in PLINK 1.9 https://www.cog-genomics.org/plink/1.9/strat#pca (cut-offs: minor allele frequency>=0.01, Hardy Weinberg Equilibrium p>= $1x10^{-6}$, pairwise SNPs $r^2$ <=0.2 in a 50kb window). The first 3 ancestry principal components (PCs) were used to correct for ancestry in the statistical model.

A linear regression model with ferritin as dependent variable with AUD and HFE (i.e., H63D-carriers *vs* wild-type) as predictor and age, BMI, sex, and ancestry PCs as covariates demonstrated a main effect of AUD on serum ferritin ($F_{1,915}$=48.5, p<0.0001). There was not a significant main effect of rs1799945 genotype ($F_{1,915}$=0.35, p=0.56) or an interaction effect of s1799945 x AUD status on serum ferritin levels ($F_{1,915}$=2.53, p=0.112).
